# Estimation of Mortality via the Neighborhood Atlas and Reproducible Area Deprivation Indices

**DOI:** 10.1001/jamanetworkopen.2025.46800

**Published:** 2026-01-12

**Authors:** Nicole Gladish, Robert L. Phillips, David H. Rehkopf

**Affiliations:** 1 Department of Epidemiology and Population Health, Stanford School of Medicine, Stanford University, Stanford, California; 2 The Center for Professionalism & Value in Health Care, Washington, DC; 3 American Board of Family Medicine, Lexington, Kentucky; 4 Division of Primary Care and Population Health, Department of Medicine, Stanford School of Medicine, Stanford University, Stanford, California; 5 Department of Pediatrics, Stanford School of Medicine, Stanford, California; 6 Department of Health Policy, Stanford School of Medicine, Stanford University, Stanford, California; 7 Department of Sociology, Stanford University, Stanford, California

## Abstract

**Importance:**

The Neighborhood Atlas Area Deprivation Index (NA-ADI) has been widely used in health policy research and incorporated into Medicare payment models such as the Accountable Care Organization Realizing Equity, Access, and Community Health model. However, calculation errors have been independently identified by multiple groups, showing that the NA-ADI contains distorted deprivation estimates that pose risks to equitable funding allocation and outcome adjustment.

**Objective:**

To develop the Reproducible ADI (ReADI) as a corrected, transparent replacement aligned with the original ADI methodology and to compare its agreement with established deprivation indices, including the Social Vulnerability Index (SVI), Social Deprivation Index, French Deprivation Index, and Neighborhood Stress Score, and its performance for estimating mortality.

**Design, Setting, and Participants:**

This cross-sectional study used 2011-2015 and 2018-2022 data from the American Community Survey (ACS), a population-based US sample across census block groups, tracts, and counties. US geographic levels with sufficient data included 235 952 block groups, 83 722 census tracts, and 3214 counties. Census tract–level 2011-2015 mortality data were obtained from the US Small-Area Life Expectancy Estimates Project. Data from the 2015 NA-ADI were accessed March 7, 2024, and data from the 2022 NA-ADI, December 1, 2024.

**Exposures:**

The ReADI was constructed using corrected methods described in the original work developing the ADI. The NA-ADI was downloaded and aggregated to tract and county levels. The SVI was from the Centers for Disease Control and Prevention. Other indices were independently constructed from the ACS.

**Main Outcomes and Measures:**

The primary outcomes were ReADI and NA-ADI comparisons against each other, other deprivation indices, and mortality.

**Results:**

The ReADI more closely aligned with other deprivation indices (*R*^2^ range, 0.609 [95% CI, 0.586-0.630] to 0.932 [95% CI, 0.931-0.933]) compared with the NA-ADI (*R*^2^ range, 0.331 [95% CI, 0.300-0.362] to 0.710 [95% CI, 0.692-0.727]). ReADI scores also better reflected component weights (*R*^2^ ≥ 0.999 [95% CI, 0.996-1.000]; RMSE ≤ 0.042 [95% CI, 0.029-0.053]) compared with the NA-ADI (*R*^2^ range, 0.832 [95% CI, 0.574-0.932] to 0.844 [95% CI, 0.601-0.937]; RMSE range, 0.346 [95% CI, 0.279-0.414] to 0.405 [95% CI, 0.334-0.470]), consistent with correct computation. In 3332 high-discrepancy census tracts representing approximately 13 million individuals, the ReADI explained more variance in life expectancy (*R*^2^ difference, 0.064; 95% CI, 0.039-0.090), particularly in underresourced urban areas.

**Conclusions and Relevance:**

In this cross-sectional study, the ReADI was correctly calculated and should replace the flawed NA-ADI for future health policy applications. Its open-source methods support transparency, adaptability, and further development.

## Key Points

**Question:**

How do calculation errors, such as failing to standardize variables, in the Neighborhood Atlas Area Deprivation Index (NA-ADI) lead to differences relative to the corrected Reproducible ADI (ReADI), and to what extent are these errors associated with mortality?

**Findings:**

In this cross-sectional study, the ReADI provided a corrected, multidimensional, and reliable measure of area deprivation, aligning better with other indices and improving estimates of association with mortality compared with the NA-ADI.

**Meaning:**

These findings suggest that the ReADI is an accurate, transparent, and policy-relevant measure that is more suitable than the NA-ADI for guiding health equity research and resource allocation.

## Teaser

This cross-sectional study evaluates the Reproducible Area Deprivation Index (ReADI) in comparison with the Neighborhood Atlas ADI other established deprivation indices for estimating mortality.

## Introduction

Since data collection on population health began, strong correlations have been observed between small-area (eg, at the census tract or block group level) health outcomes and socioeconomic characteristics, prompting efforts to systematically quantify them.^1,2^ Deprivation indices often correlate with health outcomes as strongly as individual-level socioeconomic indicators.^2,3,4,5,6^ As policy applications have grown, a range of indices have been developed internationally, including in England,^7^ New Zealand,^8^ Canada^9^ and France.^10,11^ In the US, commonly used indices include the Social Vulnerability Index (SVI),^12^ Social Deprivation Index (SDI),^13^ Neighborhood Stress Score (NSS7),^6^ and Area Deprivation Index (ADI).^14^ While these indices often share inputs and demonstrate similar associations, each was created for distinct policy or research purposes.

Among these, the ADI has been associated on a national scale with outcomes including percutaneous coronary intervention mortality,^15^ COVID-19–related mortality,^16^ diabetes outcomes,^17^ and infant brain structure.^18^ Developed by Singh in 2003 using factor analysis on 1990 US Census data,^14^ the ADI incorporated 17 socioeconomic indicators. Although frequently attributed to subsequent adaptations, Singh’s methodology^14^ remains foundational. Most recent applications use the interpretation released by Kind et al,^19^ the Neighborhood Atlas ADI (NA-ADI), derived from 5-year American Community Survey (ACS) data.^20^

However, previous studies^21,22,23,24,25^ have reported findings inconsistent with established socioeconomic patterns when applying the NA-ADI. The methods used to generate the NA-ADI are not publicly documented in sufficient detail to confirm the source of these inconsistencies, limiting transparency and the possibility of independent validation. Researchers^21,22,23,24,25^ have observed that NA-ADI scores are driven primarily by 4 of the 17 intended indicators (family income, home value, rent, and mortgage payments), the only ones not expressed as proportions. This discrepancy arises because weights derived from standardized variables in factor analysis were applied to unstandardized census data. Variables with large numerical ranges, particularly housing costs, were disproportionately amplified, distorting the intended weighting. In a reanalysis, Petterson^26^ found the correlation between an ADI constructed using unstandardized inputs and *R*^2^ values for the NA-ADI exceeded 0.9999, while correctly applying weights to standardized data reduced the value to 0.7245, demonstrating the magnitude of this flaw. Consequently, the NA-ADI functions as a biased index, particularly where housing costs diverge from other deprivation indicators. Despite repeated evaluations, it remains uncorrected.^27^

Other departures from Singh’s framework^14^ further undermine NA-ADI validity. First, it applies 1990-derived weights, despite Singh updating them for concurrent periods. Second, Singh derived weights at the tract level, yet the NA-ADI applies them at the block group level without validation. Third, aside from replacing the landline measure, thresholds have not been adjusted for inflation or economic change, such as 2022 income cutoffs remaining at less than $10 000 and $50 000 or greater.

In response, the ReADI was developed as a corrected, transparent, and methodologically updated replacement aligned with Singh’s framework.^14^ It corrects the standardization error, updates thresholds, recalculates weights using current data, and provides open documentation and code. In this cross-sectional study, the ReADI and NA-ADI are compared across geographic levels, benchmarked against other indices, and evaluated for estimation of mortality at the census-tract level. As deprivation indices enter federal payment models,^28^ reliance on a flawed index risks misallocating resources and obscuring high-need communities. A valid, transparent replacement is urgently needed.

## Methods

This cross-sectional analysis followed the Strengthening the Reporting of Observational Studies in Epidemiology (STROBE) reporting guideline for cross-sectional studies. The study used public, deidentified data, determined by the Institutional Review Board of Stanford University to exempt it from Institutional Review Board oversight and informed consent.

### Data Sources and Sample Population

Deprivation index variables used to develop the ReADI were obtained from the 2015 and 2022 US Census ACS 5-year estimates at census block group, census tract, and county levels. Data were extracted using R, version 4.5.0 (R Project for Statistical Computing),^29^ using the tidycensus package.^30^

### NA-ADI

The 2022 NA-ADI (version 4.0.1) was obtained from the Neighborhood Atlas website (accessed December 1, 2024),^31^ and the 2015 NA-ADI (version 3.1) was obtained by request (March 7, 2024). Although released only at the block group level, the NA-ADI is often applied at the tract or county level.^32,33,34,35,36^ To maintain consistency with the original multiscale approach of Singh,^14^ tract and county scores were derived by calculating population-weighted means of block group values.

### ReADI

The ReADI was developed using Singh’s methodology^14^ and updated using the 2015 and 2022 ACS data at the block group, tract, and county levels. In contrast to the NA-ADI, which suppresses certain block groups, the ReADI excluded only areas with zero population and included raw and nationally ranked scores to support researcher-defined exclusion criteria. Missing values (limited to the 4 monetary variables) were imputed using Queen contiguity-based nearest-neighbor imputation (eTable 1 in Supplement 1).^37^

Seventeen indicators were calculated as described in eTable 2 in Supplement 2. Three thresholds were updated to reflect contemporary socioeconomic conditions: (1) individuals without a high school diploma (replacing <9 years of education); (2) individuals with at least a bachelor’s degree (replacing at least a high school diploma); and (3) income disparity defined as log of 100 × ratio of households earning less than $20 000 to $100 000 or more (replacing $10 000 vs $50 000). Indicators were *z*-score standardized with log-transformed population size used as weights for factor analysis. Extracted factor scores were rescaled from 0 to 100 (higher scores indicated greater deprivation) to generate nationally ranked ReADI scores. Documentation, code, and raw scores are available elsewhere.^38^

### Other Deprivation Indices

To assess construct validity, the ReADI and NA-ADI were compared with 4 widely used deprivation indices with conceptual or methodological similarity: the SVI, SDI, NSS7, and the French Deprivation Index (FDep). The 2022 SVI was obtained from the US Centers for Disease Control and Prevention at the county and tract levels (accessed December 1, 2024) and assigned to block groups by tract. The SDI (eTable 3 in Supplement 2), NSS7 (eTable 4 in Supplement 2), and FDep (eTable 5 in Supplement 2) were calculated from 2022 ACS data using published specifications.

### Mortality Data

To assess predictive validity, publicly available all-cause mortality data from the US Small-Area Life Expectancy Estimates Project were used, providing census tract–level data for 2011 to 2015. A 2015 ReADI was generated using 2015 ACS data to align with these mortality estimates (accessed December 1, 2024).

### Statistical Analysis

To assess agreement between the ReADI and NA-ADI, linear regression was used to compare index scores at each geographic level, with *R*^2^ values, *P* values, and root mean squared errors (RMSEs) reported. NA-ADI factor weights were regressed on ReADI weights at each level. To evaluate internal consistency, Pearson correlations were calculated between each ADI and its 17 component indicators, followed by linear regression of these correlation coefficients on the original factor loadings to quantify alignment (*R*^2^ values, *P* values, and RMSEs). To assess construct validity, pairwise Pearson correlations were calculated among all 6 deprivation indices (ReADI, NA-ADI, SVI, SDI, NSS7, and FDep) across all 3 levels of geography (county, tract, and block group).

To evaluate ADI divergence, differences between the NA-ADI and ReADI were computed at each geographic level. Summary statistics (range, mean, and SD) and national county-level maps were generated. Pearson correlation was used to examine associations between score differences and the 17 component indicators.

To assess NA-ADI bias across region and urban-rural settings, census tracts and block groups were classified as urban or rural using 2020 Census Urban Area definitions. Welch *t* tests compared mean difference scores between rural and urban areas, and Cohen *d* statistic was used to quantify effect size. State-level summaries were generated by calculating the urban-rural gap as the difference in mean scores (urban-rural), with 95% CIs.

To evaluate the validity of the mortality estimates, census tracts were grouped by the absolute NA-ADI vs ReADI difference (0-9, 10-19, 20-39, and 40-100). Within each bin, linear regression was used to assess how well each ADI was associated with 2015 life expectancy. *R*^2^ values were compared using *z* tests. A sensitivity analysis adjusted for log-transformed tract population size.

All hypothesis tests were 2 sided, with significance defined as *P* < .05. Bonferroni-corrected *P* values were reported for multiple comparisons. Analyses were conducted with R, version 4.5.0 (R Project for Statistical Computing), using the stats and psych^39^ packages.

## Results

**Figure 1.**
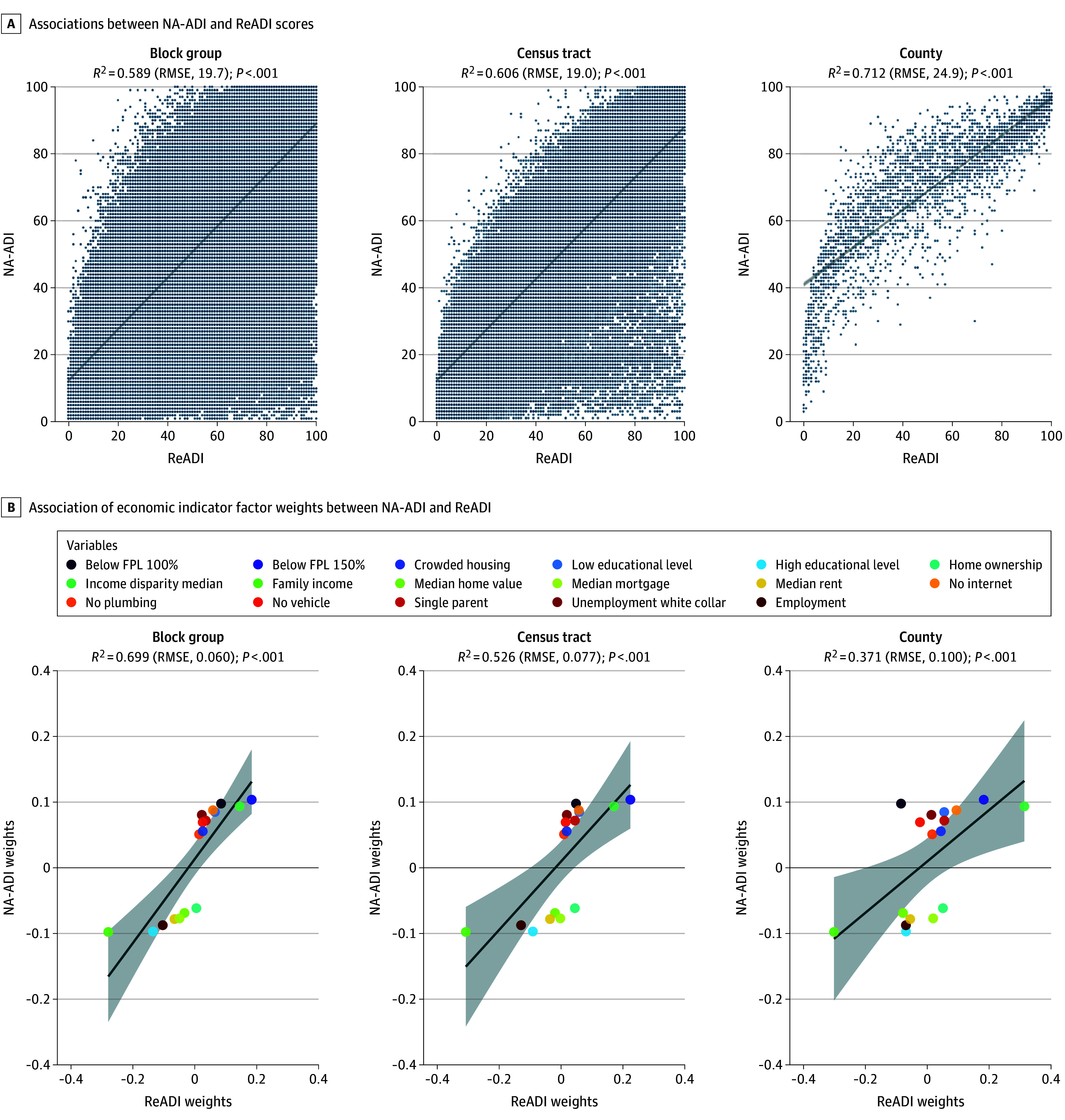
Comparison of Neighborhood Atlas Area Deprivation Index (NA-ADI) and Reproducible ADI (ReADI) Scores and Factor Weights at Multiple Geographic Scales A, Associations between NA-ADI and ReADI scores at the census block group (n = 235 952), tract (n = 83 722), and county (n = 3214) levels using linear regression (*R*^2^ values, *P* values, and root mean square error [RMSE] reported). Solid diagonal lines indicate the best linear fit between NA-ADI and ReADI values. B, Comparison of factor weights of 17 socioeconomic indicators at each geographic level using linear regression (*R*^2^ values, *P* values, and RMSE reported). Dots indicate variable weights; solid diagonal lines indicate the estimated linear relationship between NA-ADI weights and ReADI weights; shading indicates 95% CIs. FPL indicates federal poverty level.

Figure 1A shows scatterplots of the association between the NA-ADI and ReADI at 235 952 block group (column 1), 83 722 tract (column 2), and 3214 county (column 3) levels. Moderate linear associations were observed, with *R*^2^ values of 0.589 (95% CI, 0.586-0.591) for block group, 0.606 (95% CI, 0.602-0.610) for tract, and 0.712 (95% CI, 0.694-0.728) for county levels. However, discrepancies remained, with mean absolute differences of approximately 20 percentile points and RMSEs of 19.7 (95% CI, 19.6-19.8) for block group, 19.0 (95% CI, 18.8-19.1) for tract, and 24.9 (95% CI, 24.4-25.4) for county levels.

In Figure 1B, factor weights across the 17 indicators used in each index are compared. Moderate associations between weights were observed (*R*^2^ = 0.699 [95% CI, 0.337-0.872] for block group, *R*^2^ = 0.526 [95% CI, 0.132-0.785] for tract, and *R*^2^ = 0.371 [95% CI, 0.029-0.692] for county), although range differences were present where NA-ADI weights ranged from −0.098 to 0.104, while ReADI weights ranged from −0.308 to 0.314. RMSEs between weights were 0.060 (95% CI, 0.029-0.091) for block group, 0.077 (95% CI, 0.045-0.110) for tract, and 0.100 (95% CI, 0.062-0.137) for county levels, with notable differences in variables such as home ownership.

**Figure 2.**
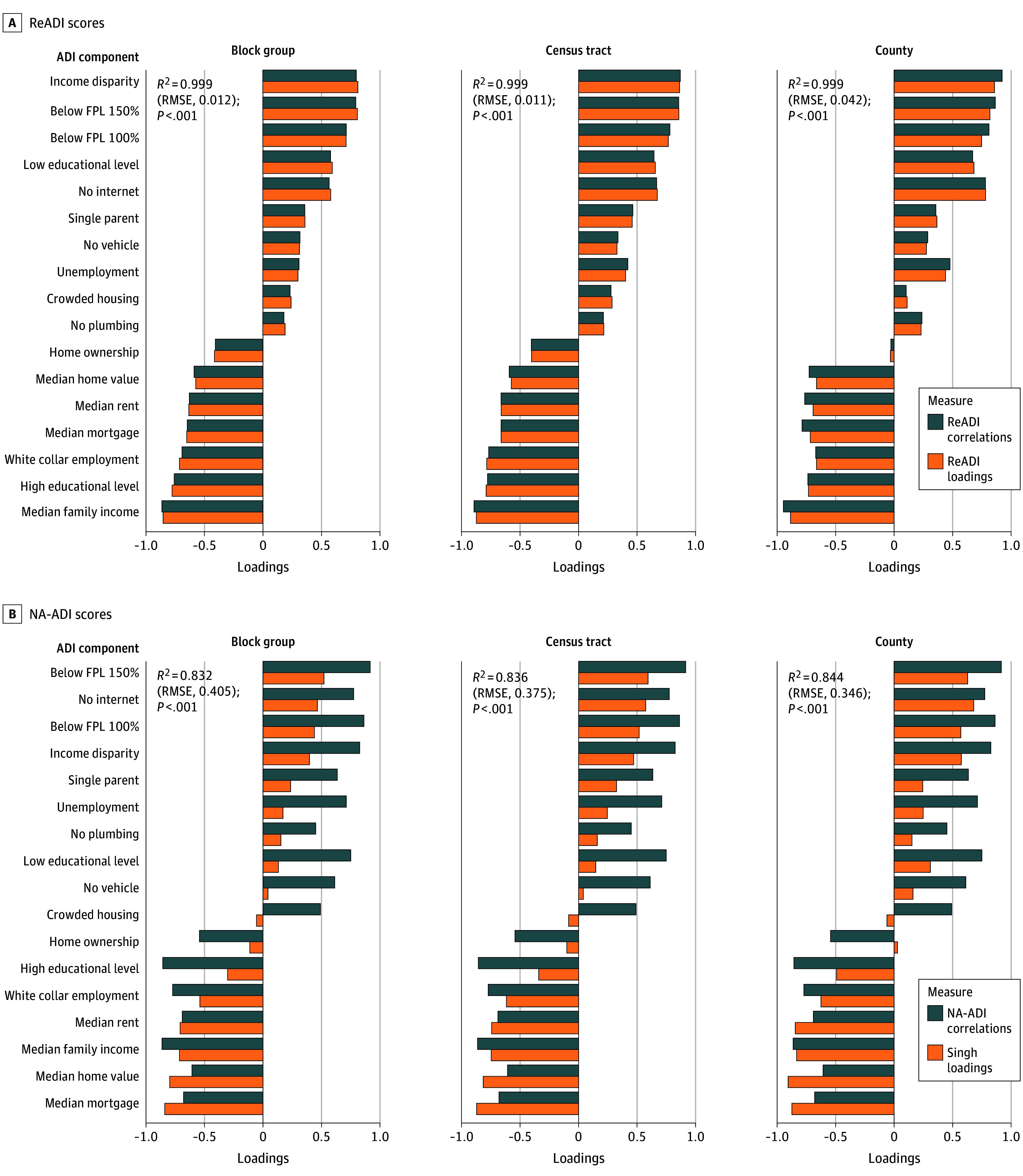
Consistency Between Area Deprivation Index (ADI) Component Correlations and Factor Loadings Across Geographic Levels A, Reproducible ADI (ReADI) scores were correlated with each socioeconomic indicator and regressed on their corresponding ReADI factor loadings (*R*^2^ values, *P* values, and root mean squared error [RMSE] reported). B, Neighborhood Atlas ADI (NA-ADI) scores were correlated with each indicator and regressed on the original factor loadings by Singh^14^ used to produce the NA-ADI. Variables are ordered by correlation values. FPL indicates federal poverty level.

Figure 2 compares each ADI’s observed correlations with its indicators and the expected contributions from factor loadings. Near-perfect alignment was demonstrated for ReADI (*R*^2^ ≥ 0.999 [95% CI, 0.996-1.000] for all geographic levels; RMSE range, 0.011 [95% CI, 0.008-0.014] to 0.042 [95% CI, 0.030-0.053]) (Figure 2A). In contrast, NA-ADI (Figure 2B) exhibited substantial misalignment (*R*^2^ range, 0.832 [95% CI, 0.574-0.932] to 0.844 [95% CI, 0.601-0.937]; RMSE range, 0.346 [95% CI, 0.280-0.416] to 0.405 [95% CI, 0.336-0.469]), with disproportionate emphasis on median home value and mortgage cost.

**Figure 3.**
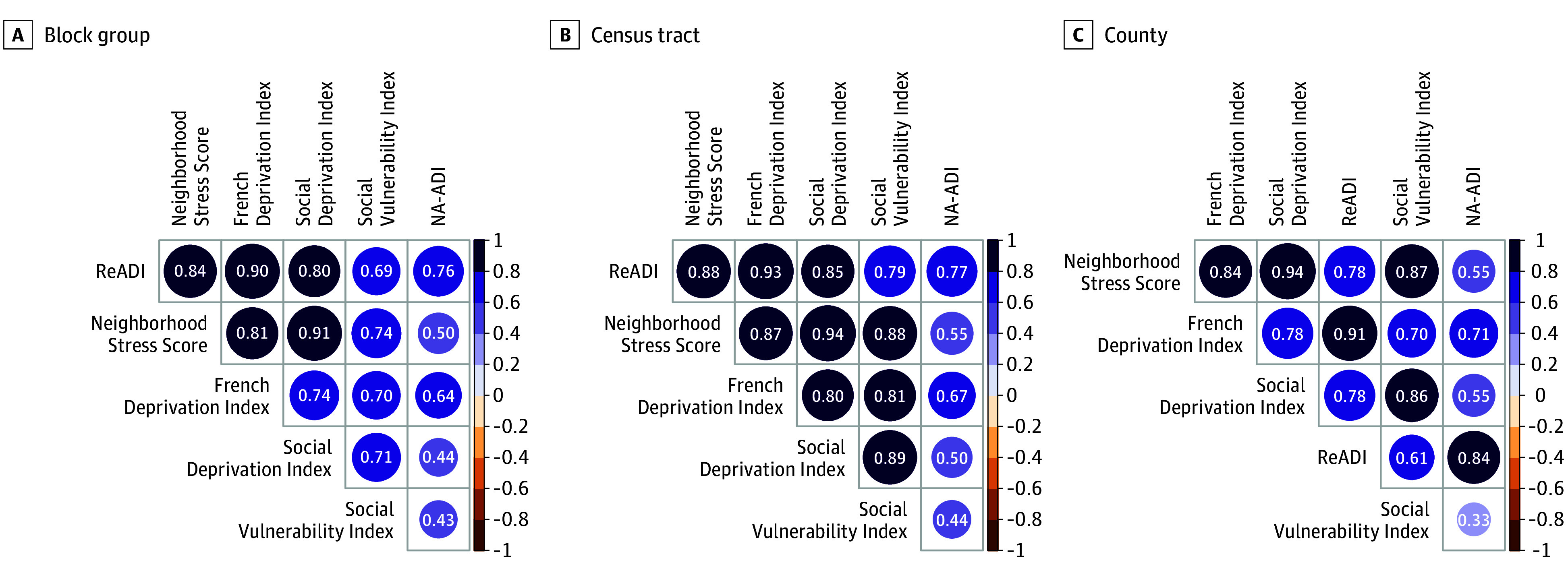
Pearson Correlations Between the Reproducible Area Deprivation Index (ReADI) and Neighborhood Atlas ADI (NA-ADI) and the 4 Established Deprivation Indices Across Geographic Levels Circle color represents correlation magnitude; circle size reflects statistical significance, with smaller circles indicating larger *P* values. Matrix order is based on the first principal component to emphasize shared variance across indices.

In Figure 3, pairwise Pearson correlation matrices compared the ReADI and NA-ADI with 4 other deprivation indices across geographic levels. Stronger correlations with other indices were consistently observed for the ReADI, with *R*^2^ values ranging from 0.609 (95% CI, 0.586-0.630) to 0.932 (95% CI, 0.931-0.933), suggesting strong convergent validity. Contrastingly, the NA-ADI demonstrated weaker correlations at each geographic level, with *R^2^* values ranging from 0.331 (95% CI, 0.300-0.362) to 0.710 (95% CI, 0.692-0.727).

**Figure 4.**
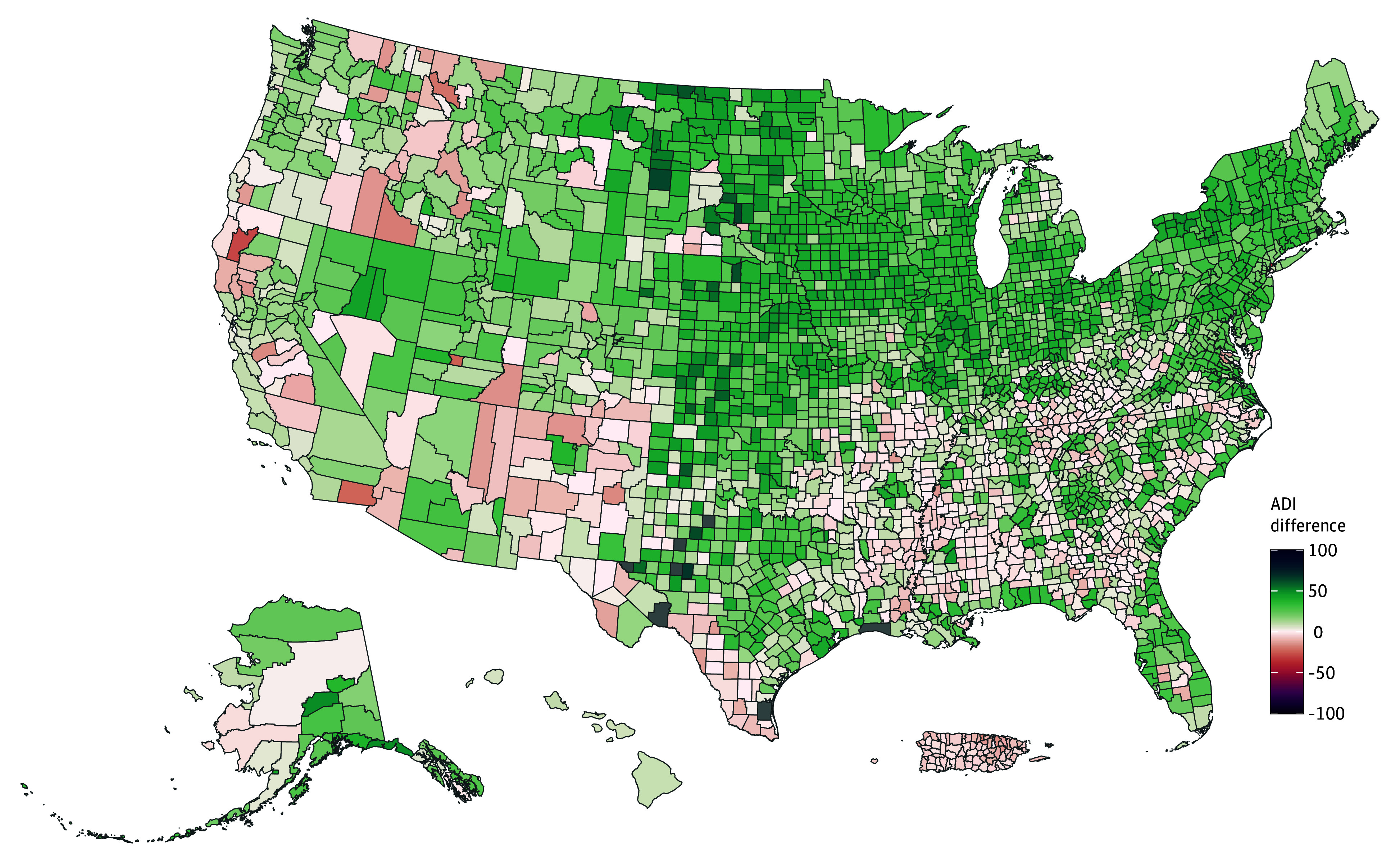
County-Level Map of Score Differences Between the Neighborhood Atlas Area Deprivation Index (ADI) and Reproducible ADI US counties are shaded according to the difference between ADIs calculated by subtracting the Neighborhood Atlas (NA-ADI) from the Reproducible ADI (ReADI). Differences range from −100 to 100, with red shading indicating counties where the NA-ADI underestimates deprivation relative to the ReADI (negative values) and green shading indicating counties where the NA-ADI overestimates deprivation (positive values).

Figure 4 maps differences between NA-ADI and ReADI scores at the county level, with green indicating higher NA-ADI scores and red indicating higher ReADI scores. eFigure 1 in Supplement 1 depicts individual ADI maps shaded by score. Mean score differences ranged from −99 to 76 percentile points at the block group (SD, 19.7), −97 to 62 at the tract (SD, 19.0), and −39 to 67 at the county level (SD, 16.4). Discrepancies of 20 points or greater were observed in 61 703 of 235 952 of block groups (26.2%), 19 705 of 83 722 tracts (23.5%), and 1579 of 3214 counties (49.1%). Rural Midwestern tracts were more often identified as deprived by the NA-ADI (5967 of 8410 [71.1%]; rural mean, 5.32 [SD, 12.13]), whereas urban deprivation tended to be underestimated (43 547 of 76 125 [57.2%]; urban mean, −0.36 [SD, 19.51]). These differences were significant across both the census tracts (rural vs urban mean, 5.68 [95% CI, 5.39-5.98]; Welch *t*, 37.7; *P* < .001) and block groups (rural mean, 5.77 [SD, 13.72]; urban mean, −0.0005 [SD, 20.30]; rural vs urban mean, 5.78 [95% CI, 5.60-5.95]; Welch *t*, 63.4; *P* < .001). Effect sizes were small to moderate across block groups (Cohen *d* = 0.29) and census tracts (Cohen *d* = 0.30), indicating differences were not driven by outliers.

Quantile regression revealed that urban-rural bias was most pronounced in more deprived areas. At the 25th percentile of deprivation, urban tracts scored 7 points lower than rural counterparts (τ = −7.0), decreasing to −3 at the median and −1 at the 75th percentile, with similar results across the block groups (τ = −8.0, −4.0, and −1.0). eFigure 2 in Supplement 1 displays state-level variation in the ADI urban-rural gap, with the largest disparities observed in New York (tract: −24.1 [95% CI, −25.4 to −22.7]; block group: −22.5 [95% CI, −23.3 to −21.7]), followed by New Jersey, Nevada, and Kansas. A minority of states, including Hawaii, Idaho, and Delaware, showed an overestimation of urban deprivation, although the magnitude was smaller.

eFigure 3 in Supplement 1 identifies the ADI components most strongly correlated with score differences, which included the proportions of residents living below 100% and 150% of the federal poverty level and those with low educational attainment across geographic levels. At the county level, income disparity and family income uniquely contributed.

**Figure 5.**
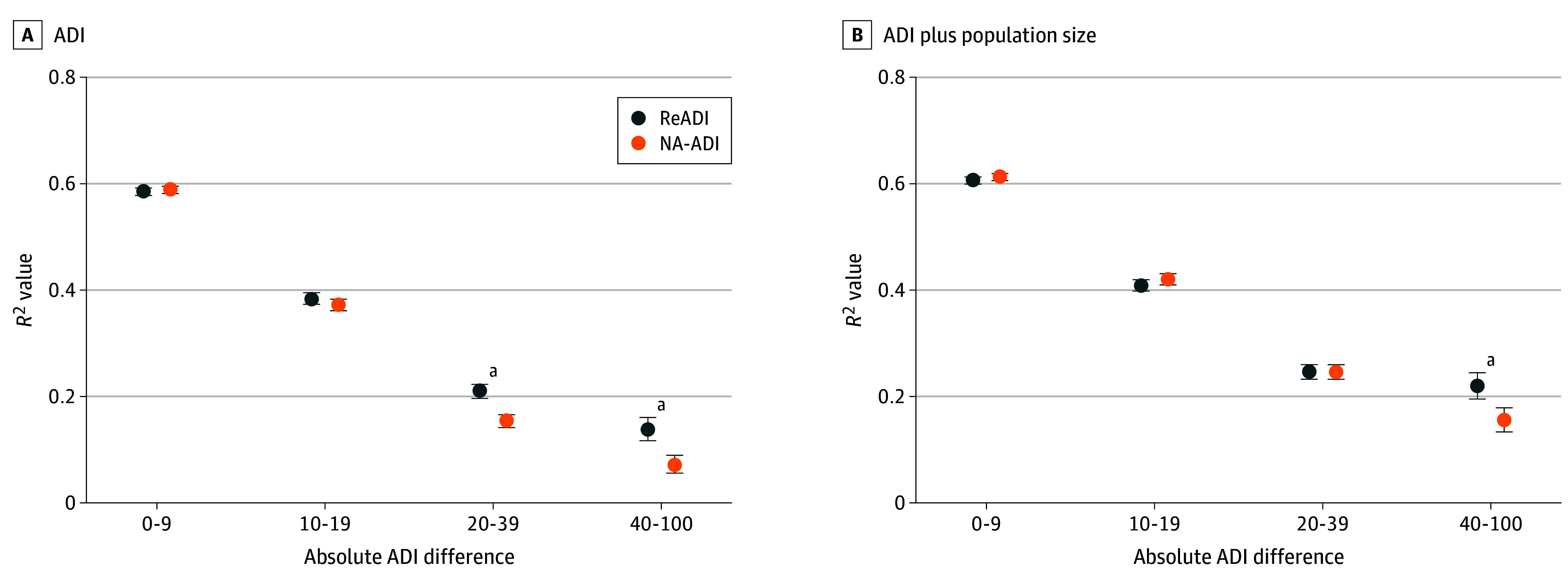
Comparison of How Each Area Deprivation Index (ADI) Is Associated With Mortality by Level of Score Disagreement Census tracts were grouped based on the absolute difference between the Neighborhood Atlas ADI (NA-ADI) and Reproducible ADI (ReADI) (0-9, 31 694 tracts; 10-19, 19 269 tracts; 20-39, 11 353 tracts; 40-100, 3332 tracts). Panels show results from linear regression models for mortality and mortality with population size. Within each group, *R*^2^ values are plotted with 95% CIs (error bars). ^a^Differences between ADIs based on Fisher *z* test were statistically significant (Bonferroni correct *P* < .0125).

Figure 5 evaluates validity for 2015 life expectancy estimates, stratified by the magnitude of NA-ADI–ReADI score disagreement. Panel A presents the unadjusted model and panel B includes population size adjustment, with statistically significant differences after Bonferroni correction. Both indices were associated with mortality across all bins (all 95% CIs excluded 0). However, the ReADI statistically outperformed the NA-ADI in tracts with score differences of 20 to 39 (*R*^2^ difference, 0.056; 95% CI, 0.040-0.073; *P* < .001) and 40 to 100 points (*R*^2^ difference, 0.066; 95% CI, 0.040-0.093; *P* = .007) (Figure 5A). In adjusted models, the ReADI remained superior in the bin of 40 or greater (*R*^2^ difference, 0.064; 95% CI, 0.039-0.090; *P* = .008) (Figure 5B). These findings suggest that errors in the NA-ADI can feasibly lead to a misallocation of resources, particularly in highly urban and deeply rural communities where misclassification is most severe.

## Discussion

This study presents the ReADI, an open-source, methodologically corrected index designed to address critical calculations errors and the structural and transparency limitations in the widely used NA-ADI.^19^ Developed in response to independent reports questioning NA-ADI’s validity, ReADI corrects calculation errors and enhances methodological consistency, transparency, and predictive performance.^14^

A key concern identified was NA-ADI’s tendency to underestimate deprivation in urban areas, particularly those with the highest observed socioeconomic disadvantage, while overestimating in many rural regions. These findings support previous observations by researchers familiar with regional socioeconomic patterns.^21^ Given the percentile-based scoring system, underestimation in one area inherently forces overestimation elsewhere, compounding the risk of inequitable resource allocation. The lack of methodological transparency in NA-ADI’s construction further exacerbates these concerns, limiting independent validation and reproducibility, both essential features for any index used in federal policy and academic research.

Beyond correcting the standardization error identified by Petterson,^26^ the ReADI incorporates updated indicator thresholds, recalculates factor loadings using contemporaneous census data, applies population-weighted adjustments, and generates scores independently at block group, tract, and county levels. Unlike the NA-ADI, the ReADI provides full transparency, offering raw scores, documentation, and code to enable custom thresholds and independent validation.

The moderate correlation between the ReADI and NA-ADI underscores the cumulative impact of the methodological differences. The ReADI demonstrated internal consistency between its factor loadings and component indicators, as expected when weights are applied correctly. The NA-ADI, by contrast, showed markedly weaker alignment, indicating that its published scores deviate from intended weighting and violate fundamental principles of composite index construction. This discrepancy reflects mathematical inconsistency rather than methodological preference: correctly applied factor-derived weights must, by definition, yield composites strongly correlated with their inputs. NA-ADI’s failure to meet this standard raises serious concerns about its internal validity and policy application.

Although many deprivation indices capture related aspects of socioeconomic disadvantage, they were developed for different policy, surveillance, or research purposes. Indices such as the SDI,^13^ SVI,^12^ NSS7,^6^ and FDep^11^ differ in construction, intent, and application. Nonetheless, comparison with ReADI is appropriate, as all quantify some type of contextual disadvantage and inform health equity research. Across these comparisons, the ReADI consistently demonstrated stronger correlations across all geographic levels, whereas the NA-ADI remained a consistent outlier. Importantly, the ReADI is not simply another deprivation index; it is a direct methodological correction to the Neighborhood Atlas implementation of the ADI, which diverged from Singh’s original specifications.^14^ The ReADI adheres to Singh’s factor-analytic framework while updating thresholds when applying contemporary data. Given NA-ADI’s widespread policy and research use, the ReADI was designed to serve as its transparent, corrected, and publicly accessible replacement.

These discrepancies have direct implications when deprivation indices guide medical payment models or resource allocation. The largest divergences between the ReADI and NA-ADI occurred in rural areas, especially in the Midwest, where the NA-ADI frequently assigns inflated deprivation scores. This overestimation likely reflects the standardization error identified by Petterson,^26^ wherein unstandardized variables with large values (eg, home value, mortgage cost) dominate the composite. Consequently, low-cost rural areas are misclassified as highly deprived, while high-cost urban areas are underweighted, despite other indicators. This pattern diverges from Singh’s original framework,^14^ which emphasized poverty-related measures such as the proportion of residents below 150% of the federal poverty line. The indicators most strongly associated with NA-ADI–ReADI differences were also the most heavily weighted in Singh’s analysis, underscoring that NA-ADIs structural miscalculations introduce systemic bias.

This conclusion is reinforced by the mortality analysis, where the ReADI explained greater variance in life expectancy than the NA-ADI, particularly in tracts with score differences of 40 or more points. These 3332 census tracts represent an estimated 13 million individuals, illustrating that the NA-ADI’s flaws are not theoretical but materially distort policy and resource decisions. The ReADI’s validity for mortality estimates has also been demonstrated in a separate nationwide study,^40^ where it outperformed or matched other indices across outcomes, including diabetes control, cancer screening, and behavioral health. Together, these findings confirm ReADI’s broader applicability as a health-relevant deprivation metric and its reliability as a tool for guiding public health strategies, particularly where NA-ADI misclassification is most severe. Confidence in deprivation indices is essential for equitable payment models, yet the NA-ADI prompted ad hoc adjustments such as scale modifications in the accountable care organization Flex model.^24^ The Centers for Medicare & Medicaid Services have since developed a revised index, but it remains a closed source.^41^

### Limitations

This study has several limitations that should be noted. First, because the NA-ADI is released only at the block group level, population-weighted aggregation was used to estimate tract and county scores. While common in area-based research, this aggregation may introduce errors in geographically heterogenous areas. Second, mortality validation was limited to all-cause mortality from 2011 to 2015, the only nationally representative small-area mortality data that were publicly available. The absence of more recent public data restricts assessment of current outcomes and reflects a broader challenge in disparities research. Third, our results were not stratified by race or ethnicity, an important direction given the intersections of structural racism, geography, and socioeconomic deprivation. Future work will extend ReADI validation to additional outcomes and populations, reexamine studies that relied on the NA-ADI, and expand collaborations with agencies such as the US Census Bureau. Transparent, methodologically sound, and up-to-date indices are essential for advancing equity in health research and policy.

## Conclusions

In this cross-sectional study, the ReADI represented the correct implementation of the ADI, addressing structural errors in the NA-ADI. The NA-ADI’s flawed construction produced substantial misclassification, undermining research validity and equity-focused policy. In contrast, the ReADI adhered to Singh’s factor-analytic framework while enhancing transparency, accessibility, and predictive accuracy, including improved mortality prediction. The ReADI offers an open, replicable alternative aligned with health equity goals.

## Notes

Supplement 1. **eTable 1.** Number of Missing Variables at the Census Block Group, Tract, and County Levels for the ReADI Created Using 2022 5-Year ACS Data **eFigure 1.** Map of US Counties Shaded by Scores for the ReADI and the NA-ADI **eFigure 2.** Mean Difference Between the Neighborhood Atlas (NA-ADI) and Reproducible Area Deprivation Index (ReADI) by State, Regions, and Area Type **eFigure 3**. Correlations Between Each Indicator With the ADI Difference Scores Across Geographies

Supplement 2. **eTable 2.** Data Dictionary, Census Tables, Formulas, Weights, and Loadings for the 2022 and 2015 Reproducible ADI **eTable 3.** Data Dictionary, Census Tables, and Formulas for the 2022 Social Deprivation Index (SDI) **eTable 4.** Data Dictionary, Census Tables, and Formulas for the 2022 Neighborhood Stress Score (NSS7) **eTable 5.** Data Dictionary, Census Tables, and Formulas for the 2022 US Adapted French Deprivation Index

Supplement 3.
Data Sharing Statement
