# Supplementary material for: Estimation of Mortality via the Neighborhood Atlas and Reproducible Area Deprivation Indices
Source: JAMA Netw Open. 2026 Jan 12;9(1):e2546800. doi: 10.1001/jamanetworkopen.2025.46800 (PMC12797101; doi:10.1001/jamanetworkopen.2025.46800)
Supplement: Supplement 1. — eTable 1. Number of Missing Variables at the Census Block Group, Tract, and County Levels for the ReADI Created Using 2022 5-Year ACS Data eFigure 1. Map of US Counties Shaded by Scores for the ReADI and the NA-ADI eFigure 2. Mean Difference Between the Neighborhood Atlas (NA-ADI) and Reproducible Area Deprivation Index (ReADI) by State, Regions, and Area Type eFigure 3. Correlations Between Each Indicator With the ADI Difference Scores Across Geographies [file jamanetwopen-e2546800-s001.pdf]

## Supplementary Online Content

Gladish N, Phillips RL, Rehkoph DH. Estimation of mortality via the Neighborhood Atlas and Reproducible Area Deprivation Indices. *JAMA Netw Open.* 2025;8(12):e2546800. doi:10.1001/jamanetworkopen.2025.46800

**eTable 1.** Number of Missing Variables at the Census Block Group, Tract, and County Levels for the ReADI Created Using 2022 5-Year ACS Data

**eFigure 1.** Map of US Counties Shaded by Scores for the ReADI and the NA-ADI

**eFigure 2.** Mean Difference Between the Neighborhood Atlas (NA-ADI) and Reproducible Area Deprivation Index (ReADI) by State, Regions, and Area Type

**eFigure 3.** Correlations Between Each Indicator With the ADI Difference Scores Across Geographies

This supplementary material has been provided by the authors to give readers additional information about their work.

© 2025 Gladish N et al. *JAMA Network Open*.

**eTable 1. Number of missing variables at the census block group, tract, and county levels for the ReADI created using 2022 5-year ACS data. Abbreviations: ReADI, Reproducible Area Deprivation Index; ACS, American Community Survey.**

| Variable | Block Group N (%) | Census Tract N (%) | County N (%) |
|---|---|---|---|
| Median Income, $ | 15,042 (6) | 724 (1) | 1 (<1) |
| Median Gross Rent, $ | 65,920 (27) | 4,392 (5) | 10 (<1) |
| Median Home Value, $ | 21,260 (9) | 2,344 (3) | 3 (<1) |
| Median Monthly Mortgage, $ | 29,910 (12) | 2,796 (3) | 6 (<1) |

**eFigure 1. U.S. county-level deprivation scores for the Neighborhood Atlas ADI and Reproducible ADI.**

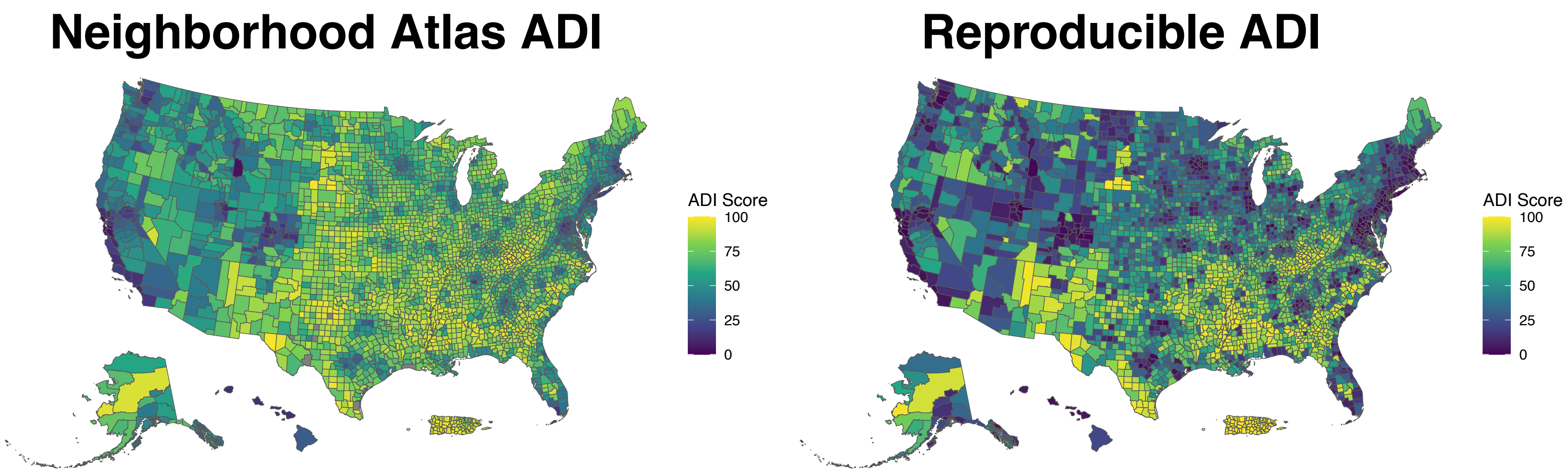


Scores range from 0 (least deprived, dark blue) to 100 (most deprived, light green).
**Abbreviations:** ADI, Area Deprivation Index

**eFigure 2. Mean difference between the Neighborhood Atlas (NA-ADI) and Reproducible Area Deprivation Index (ReADI) by state, regions, and area type.**

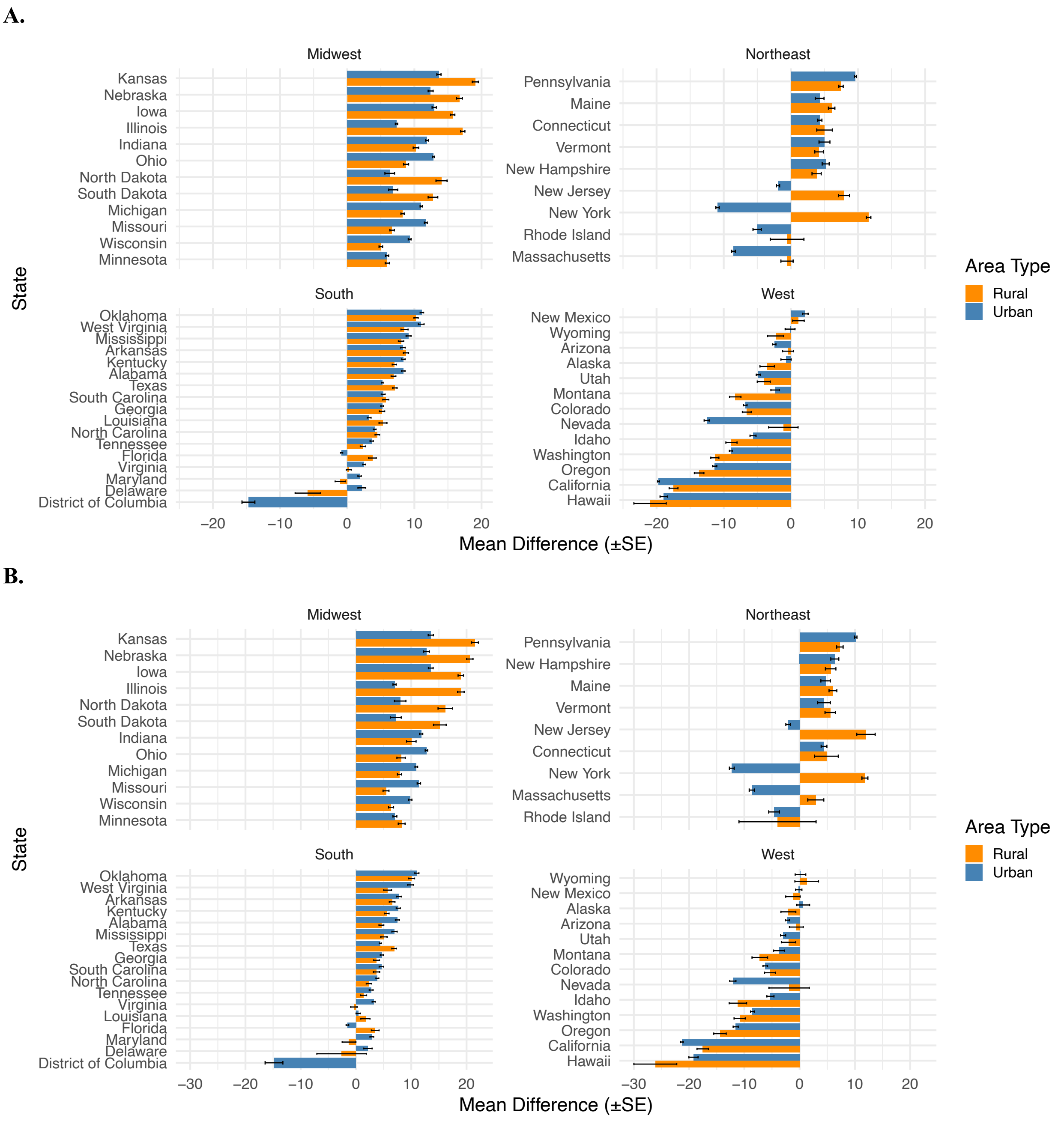


Bars represent the mean difference stratified by rural versus urban classification and grouped by U.S. Census region at the census block group (A) and tract (B) level. Positive values indicate greater deprivation estimated by the NA-ADI relative to the ReADI; negative values indicate lower deprivation. Error bars represent standard errors of the mean. Urban-rural status was assigned based on 2020 Census Urban Area boundaries.
**Abbreviations:** SE, standard error.

**eFigure 3. Correlations between socioeconomic indicators and the difference between the Neighborhood Atlas (NA-ADI) and the Reproducible Area Deprivation Index (ReADI) across geographies.**

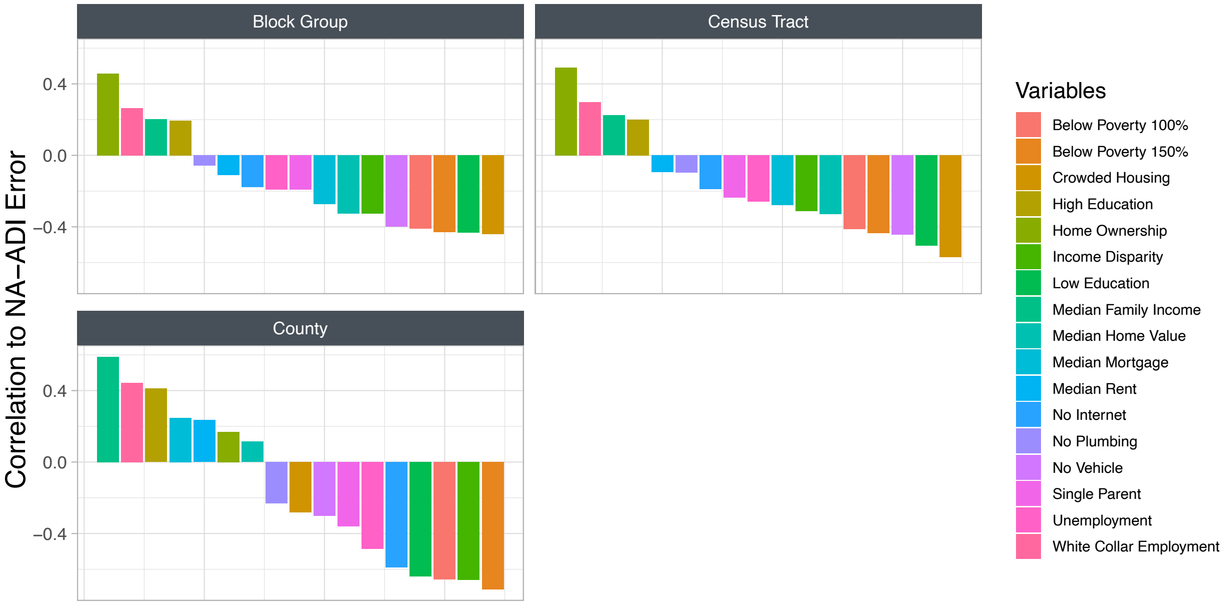


Difference scores were calculated as NA-ADI - ReADI. Pearson correlations were computed between each indicator and the difference scores. All associations were statistically significant after Bonferroni correction (*P* <.001).
