# Supplementary material for: Estimation of Mortality via the Neighborhood Atlas and Reproducible Area Deprivation Indices
Source: JAMA Netw Open. 2026 Jan 12;9(1):e2546800. doi: 10.1001/jamanetworkopen.2025.46800 (PMC12797101; doi:10.1001/jamanetworkopen.2025.46800)
Supplement: Supplement 3. — Data Sharing Statement [file jamanetwopen-e2546800-s003.pdf]

Data Sharing Statement

Gladish. Estimation of Mortality via the Neighborhood Atlas and Reproducible Area Deprivation Indices. *JAMA Netw Open*. Published December 08, 2025.
doi:10.1001/jamanetworkopen.2025.46800

Data
Data available: Yes
Data types: Deidentified participant data
How to access data: All code and data for the analysis will be made publicly available.
When available: With publication

Supporting Documents
Document types: Statistical/analytic code
How to access documents: https://sepi.sites.stanford.edu/resources
When available: With publication

Additional Information
Who can access the data: Anyone requesting the data.
Types of analyses: Any purpose.
Mechanisms of data availability: Available for download by anyone.
